# A Multicomponent Intervention (POSSIBLE) to Improve Perceived Risk for HIV Among Black Sexual Minority Men: Feasibility and Preliminary Effectiveness Pilot Study

**DOI:** 10.2196/54739

**Published:** 2024-06-11

**Authors:** Derek T Dangerfield II, Janeane N Anderson, Charleen Wylie, Ricky Bluthenthal, Chris Beyrer, Jason E Farley

**Affiliations:** 1 Milken Institute School of Public Health George Washington University Washington, DC United States; 2 University of Tennessee Health Science Center Memphis, TN United States; 3 Keck School of Medicine of USC University of Southern California Los Angeles, CA United States; 4 Duke Global Health Institute Durham, NC United States; 5 Johns Hopkins School of Nursing Baltimore, MD United States

**Keywords:** pre-exposure prophylaxis, PrEP, sexual health, peers, apps, community, mobile phone, HIV, sexual minority, minority communities, minority, Black, African American, peers, patient education, self-monitoring, treatment adherence, treatment participation, community, community health, mobile health, digital health, digital technology, digital interventions, smartphones

## Abstract

**Background:**

Increased pre-exposure prophylaxis (PrEP) use is urgently needed to substantially decrease HIV incidence among Black sexual minority men. Low perceived risk for HIV (PRH) is a key unaddressed PrEP barrier for Black sexual minority men. Peers and smartphone apps are popular intervention tools to promote community health behaviors, but few studies have used these together in a multicomponent strategy. Therefore, we designed a multicomponent intervention called POSSIBLE that used an existing smartphone app called PrEPme (Emocha Mobile Health, Inc) and a peer change agent (PCA) to increase PRH as a gateway to PrEP.

**Objective:**

This paper aims to describe the feasibility and preliminary impact of POSSIBLE on PRH and willingness to accept a PrEP referral among Black sexual minority men.

**Methods:**

POSSIBLE was a theoretically guided, single-group, 2-session pilot study conducted among Black sexual minority men from Baltimore, Maryland between 2019 and 2021 (N=69). POSSIBLE integrated a PCA and the PrEPme app that allows users to self-monitor sexual risk behaviors and chat with the in-app community health worker to obtain PrEP service information. PRH was assessed using the 8-item PRH scale before and after baseline and follow-up study visits. At the end of each study visit, the PCA referred interested individuals to the community health worker to learn more about PrEP service options.

**Results:**

The average age of participants was 32.5 (SD 8.1, range 19-62) years. In total, 55 (80%) participants were retained for follow-up at month 1. After baseline sessions, 29 (42%) participants were willing to be referred to PrEP services, 20 (69%) of those confirmed scheduled appointments with PrEP care teams. There were no statistically significant differences in PRH between baseline and follow-up visits (*t*_122_=–1.36; *P*=.17).

**Conclusions:**

We observed no statistically significant improvement in PRH between baseline and month 1. However, given the high retention rate and acceptability, POSSIBLE may be feasible to implement. Future research should test a statistically powered peer-based approach on PrEP initiation among Black sexual minority men.

**Trial Registration:**

ClinicalTrials.gov NCT04533386; https://clinicaltrials.gov/study/NCT04533386

## Introduction

Increased pre-exposure prophylaxis (PrEP) use is urgently needed to substantially decrease HIV incidence among Black sexual minority men who have an estimated 50% lifetime risk of HIV acquisition [1]. Data show racial inequities in uptake and adherence among sexual minority men who meet PrEP indications [2]. Socioecological factors such as side effect concerns, stigma, low health care access, and poor clinical experiences including patient-clinician communication remain substantial PrEP barriers for this community [3-5]. Low perceived risk for HIV (PRH) is also a key unaddressed PrEP barrier [6-9]. Some Black sexual minority men have low concerns about HIV acquisition because they think their current behaviors are lower risk than their past or their peers’ behaviors and they do not associate HIV status with quality of life [6,10]. Other reasons for low PRH include being in a monogamous relationship and limited sexual activity [6,9,11]. However, low PRH inadequately reflects objective risks and disease severity for Black sexual minority men, given the high-community HIV incidence, suboptimal HIV care outcomes, and negative health consequences of long-term infection [7,8]. Therefore, multicomponent interventions that address PRH and other known barriers are needed to improve PrEP uptake.

Some HIV prevention interventions leverage in-group members as peer change agents (PCAs) to disseminate health-related information within the community for behavior change [12-15]. Peers are considered a valuable resource in marginalized communities to obtain health information, discuss taboo experiences, and help group members understand why behavior change could be beneficial [6,16,17]. Peers can facilitate behavior change because they have similar experiences, can address social barriers, and can improve health literacy [12,13,16,18]. PCAs are uniquely positioned to influence health behaviors because their roles as community members, patients, and health care paraprofessionals can build trust and lead others to credible information or clarify the information [17]. PCAs have improved behavioral health [12], HIV testing [19], medication adherence, and PrEP [13-15] for HIV prevention and could be effective interventionists among Black sexual minority men.

Other interventions have used smartphone apps as electronic diaries to reduce sexual risks through self-monitoring behaviors, which facilitates reflection [20-22]. Technology-based interventions could also be effective for Black sexual minority men because many of them use apps and other mobile devices for several purposes, including partner seeking, social network development, and health information [23,24]. Using apps for interventions could help Black sexual minority men circumvent the social and structural barriers to PrEP such as perceived judgment, stigma, and discrimination from clinicians. Since peers and smartphone apps are typically used independently in interventions, they could have a stronger impact if combined into a multicomponent health communication strategy because they could hypothetically reduce socioecological barriers for Black sexual minority men simultaneously.

Motivational interviewing (MI) is a communication approach in which a professional collaborates with individuals to activate their motivations to change behavior [17,25]. Some studies use “motivational interview consistent” interventions for HIV prevention because of their cost-effectiveness, brevity, and use of client interests for behavior change [26]. However, existing MI-based interventions to improve PRH or PrEP among Black sexual minority men are limited [18]. Additionally, factors known to drive PRH or PrEP use among Black sexual minority men by PCAs may not be fully leveraged in traditional MI-based interventions [27].

We designed a multicomponent intervention called POSSIBLE that used a PCA and an existing smartphone app called PrEPme (Emocha Mobile Health, Inc) [25] to increase PRH as an important cognitive gateway to PrEP uptake among Black sexual minority men. The intervention was guided by life course theory [28,29], the health belief model [30,31], and possible selves [6,32]. Life course theory suggests that timing, major life events, and age-related vulnerabilities impact sexual health behaviors [28,33]. The health belief model posits that risk perceptions catalyze behavior change [9,30]. Possible selves suggests that ideas of what individuals could or want to become can influence behavior [6,32,34]. Taken together, we hypothesized that a PCA who represented a “future self” could influence PRH at particular points along the life course of Black sexual minority men by cueing individuals to action through a review of their sexual risks in PrEPme and successfully encourage others to use PrEP having navigated similar social challenges [6].

This paper describes the feasibility and preliminary effects of POSSIBLE on PRH and subsequent willingness to accept a PrEP referral among Black sexual minority men. More information on the feasibility and effects of a multicomponent strategy using a PCA and smartphone app is needed to advance the promise of their combined effectiveness. Given the extreme racial disparity in PrEP use among sexual minority men [35,36], strategies that can increase PrEP uptake are still needed. Findings will provide insights into the usefulness of combining 2 popular HIV prevention interventions into a multicomponent strategy and elucidate the cognitive and cultural aspects of health decision-making among this vulnerable community.

## Methods

### Ethical Considerations

All study procedures were approved by the Johns Hopkins School of Medicine Institutional Review Board (IRB00241244). Oral informed consent was audio-recorded and documented in study folders prior to conducting baseline study visits. Participants were given study ID numbers, and identifiable information (ie, names and data) was stored on private, password-protected servers. Participants were provided a US $50 electronic Amazon gift card for the baseline visit.

### Study Design

POSSIBLE was a single-group, 2 session pilot intervention conducted between 2019 and 2021 that was refined using the ADAPT-ITT (Assessment, Decision, Administration, Production, Topical Experts, Integration, Training, and Testing) model [37]. Formative research was conducted to refine key aspects of the intervention approach such as the usefulness of the app-based diary and PCA characteristics among Black sexual minority men of different age groups [6].

### Peer Change Agent

POSSIBLE incorporated a PCA who was matched with participants’ key demographic and cultural characteristics as guided by theory, previous studies, and formative research identifying preferences among Black sexual minority men [6,38]. Specifically, studies suggested that the PCA should have similar experiences as Black sexual minority men (eg, navigating interactions with romantic or sexual partners, clinicians, and health insurance) and be a “future self” to whom they could aspire [6,38]. Therefore, the principal investigator (PI to DTD), used Descovy for PrEP and served the intervention as the PCA. Further details regarding the experience and dual role of the PI serving as a PrEP-using PCA have been published in an autoethnography [17].

### PrEPme Smartphone App

PrEPme was designed for Maryland users to obtain statewide PrEP service information and navigation support from an app-based community health worker (CHW) [25]. PrEPme also allows users to self-monitor sexual risk behaviors, view a graph of sexual risk behaviors by week and month, and chat with the CHW in the app to obtain PrEP service information [6].

### Linkage to PrEP Care

A CHW supervised by a nurse case manager within the Center for Infectious Disease and Nursing Innovation at the Johns Hopkins School of Nursing (previously known as the REACH Initiative) provided navigation services including reviewing eligibility for or access to medical insurance, identifying preferred clinic locations, and arranging appointment and scheduling activities. Occasionally, the PCA referred participants or helped schedule appointments at PrEP service organizations for individuals who wanted to avoid interfacing with additional staff associated with a medical research institution.

### Study Procedures

#### Study Enrollment

A research assistant screened individuals who were interested in the study for eligibility via phone. Eligible individuals were emailed an informed consent form, details regarding their scheduled web-based baseline appointment, and an electronic survey assessing demographic and behavioral characteristics and PRH. Individuals were given the opportunity to ask the research team (including the PI) questions regarding the study via phone or email prior to their baseline visit.

#### Web-Based Baseline Study Visit

Due to COVID-19, baseline and follow-up study visits were conducted via Zoom (Zoom Video Communications). Prior to the baseline visit, the PI or PCA provided participants an additional opportunity to ask questions regarding the study and obtained oral informed consent via Zoom [39]. The script guided the PCA to obtain information regarding participants’ lifestyles, personal goals and values, relative HIV risks, and PRH, then tailor health communication based on their responses to influence PRH and encourage PrEP use regardless of participants’ reported behaviors [16,26,27,29]. Example questions included, “How would being diagnosed with HIV impact your goals?” and “Given that research suggests that 50% of Black sexual minority men will get HIV despite the fact that they use condoms more than other people, how likely do you think you will get it?” [6,40]. The script also provided opportunities for the PCA to address HIV or PrEP misinformation and disclose PrEP use to share experiences managing potential side effects, challenges disclosing use to romantic or sexual partners, and empathy regarding participants’ stigmatizing clinical experiences [17,40].

At the end of the session, the PCA referred interested individuals to the CHW as described earlier. Individuals who declined referrals to the CHW were provided alternative service locations for PrEP care and referred upon request. Baseline visits lasted between 45 and 60 minutes (accounting for informed consent, rapport building, 15- to 20-minute conversation, and PrEP navigation for those who were interested), at the end of which participants were asked to download PrEPme to record their sexual risk behaviors in its app-based diary for 1 month. Baseline study visit procedures and effects have been published [40].

#### Web-Based Follow-Up Visit

In the second session, the PCA reviewed the PrEPme diary with participants and conducted another MI-consistent conversation to explore relative HIV risk behaviors, review behavioral alignment with goals and values, and reassess their PrEP interests. At the end of the session, the PCA referred interested individuals to the CHW as described earlier. Individuals who declined referrals to the CHW were provided alternative service locations for PrEP care and referred upon request. Follow-up visits lasted between 20 and 30 minutes, and participants were provided another US $50 electronic Amazon gift card for completing follow-up visits regardless of reported app use.

#### PrEP Referral

All participants were first referred a CHW at the Johns Hopkins School of Nursing who could help navigate them to PrEP services. Participants who were interested in case management from the CHW were linked to services of their choice. Individuals who declined referrals to the CHW were offered direct referrals by the PCA who reached out to the requested case management services to help schedule appointments.

#### Satisfaction Surveys

Participants completed a satisfaction survey that also assessed their PRH at the end of the baseline session prior to downloading PrEPme, then again at the end of their follow-up appointment.

### Participants

Participants were recruited using a combination of active and passive strategies [39,41] in Baltimore, Maryland, and were eligible based upon the following self-reported criteria: Black or African American race, identifying as a cisgender person, being 18 years and older of age, same-sex attraction to men, HIV-negative, and having oral or anal sex with ≥1 male partner in the previous 6 months.

### Measures

This concept was assessed using the 8-item PRH scale from Napper et al [42] before and after the baseline visit. Sample questions included items assessing concerns about HIV and perceived likelihoods of infection. Total possible scores ranged from 10 to 40, higher scores indicate greater PRH. The scale was found to be reliable (8 items, α=.78).

### Data Analysis

Paired 1-tailed *t* tests were used to examine changes in PRH after the end of the study. Descriptive statistics were used to explore the proportion of participants who were referred to services and scheduled a PrEP appointment after baseline.

## Results

A total of 291 individuals were screened for the study, 93 of whom were eligible. Among eligible individuals, 69 participated and 55 (80%) were retained for follow-up at month 1. Table 1 describes the sociodemographic characteristics and PrEP referral willingness among participants. The average age of participants was 32.5 (SD 8.1, range 19-62) years. Additionally, 52 (75%) identified as gay, 11 (16%) identified as bisexual, 51 (74%) reported being employed full-time or part-time at baseline, 58 (84%) reported having insurance coverage, 54 (78%) reported being single, and 32 (47%) reported ever having a sexually transmitted infection. After baseline sessions, 29 (42%) participants were willing to be referred to PrEP services, and 20 (69%) of them confirmed scheduled appointments with PrEP care teams.

Regarding the use of the mobile app–based diary, 17 (31%) follow-up participants reported recording an entry every week prior to their follow-up appointment, 3 (5%) reported using the app for half of the weeks, and 6 (11%) reported that they did not use the app at all. In total, 11 (20%) reported initiating PrEP prior to follow-up, and 15 (27%) of follow-up participants were willing to be referred to PrEP services. There were no statistically significant differences in mean PRH scores between baseline (21.2, SD 5.5) and follow-up (23.6, SD 5.7) visits (t_122_=–1.36; *P*=.17).

**Table 1:** Sociodemographic characteristics and PrEP^a^ referral willingness among Black sexual minority men in POSSIBLE 2019-2021 (N=69).

		Baseline	Month 1 follow-up
**Age (years)**			
	Mean (SD)	32.5 (8.1)	32.7 (7.7)
	Range	19-62	19-50
	<35 years, n (%)	49 (71)	40 (73)
**Sexual orientation, n (%)**			
	Homosexual, gay, same gender-loving	52 (75)	42 (76)
	Bisexual	11 (16)	7 (13)
	Other	6 (9)	6 (11)
**Employment status, n (%)**			
	Full-time	44 (64)	36 (65)
	Part-time	7 (10)	6 (11)
	Unemployed	13 (19)	11 (20)
	Other	5 (7)	2 (4)
**Highest level of education, n (%)**			
	Grade 11 or less	5 (7)	1 (2)
	Grade 12 or GED^b^	10 (14)	11 (20)
	Associate degree	2 (3)	0 (0)
	Some college	10 (14)	7 (13)
	Bachelor degree	24 (35)	16 (29)
	More than bachelor degree	18 (26)	20 (36)
Health care coverage, n (%)		58 (84)	51 (93)
**Annual gross income (US $), n (%)**			
	Less than $20,000	15 (22)	15 (27)
	Between $30,000 and $40,000	8 (11)	12 (22)
	Between $40,000 and $50,000	9 (13)	7 (13)
	Between $50,000 and $60,000	6 (9)	4 (7)
	More than $60,000	23 (33)	17 (31)
**Relationship status, n (%)**			
	Single or not in a relationship	54 (78)	45 (82)
	In a committed relationship	9 (13)	7 (13)
	Married	2 (3)	0 (0)
	Other	2 (3)	3 (5)
STI^c^ history past 6 months, n (%)		32 (47)	42 (76)
Drug use before sex past 6 months, n (%)		47 (68)	39 (70)
**Willingness to be referred to PrEP services, n (%)**		29 (42)	15 (27)
	PrEP appointment scheduled	20 (69)	—^d^
	Initiated PrEP prior to month 1	—	11 (20)

^a^PrEP: pre-exposure prophylaxis.

^b^GED: General Educational Diploma.

^c^STI: sexually transmitted infection.

^d^Not available.

## Discussion

### Principal Findings

This study explored the feasibility and preliminary effectiveness of POSSIBLE, a multicomponent intervention using a PCA and mobile app–based diary to improve PRH among Black sexual minority men. Given the high retention rate, POSSIBLE may be a feasible multicomponent strategy to implement among Black sexual minority men. We found improvements in PRH after baseline sessions [40]. However, we observed no statistically significant improvement in PRH after intervention from baseline to month 1.

We observed relatively low PRH scores at baseline and month 1 follow-up. Analyses showed that the PCA increased baseline PRH scores [40]. The effects of the intervention may have been maximized in the baseline session such that the addition of the app for reflexivity could not increase scores from baseline to month 1. Other studies have found that competing survival priorities supersede HIV-related concerns in the lives of Black sexual minority men. The shift from HIV as a “death sentence” in the early days of the epidemic to its positioning as a manageable chronic health condition could be a key reason for low PRH and for why perceived risk did not change from baseline to month 1. Black sexual minority men may also consider their current behaviors relatively safer than their past or their peers’ behaviors as found in previous studies [6,10]. Some may appraise their vulnerability based upon their most recent behaviors, which may not have involved condomless sex or drug use in the month of the intervention, which was conducted partly during the height of the global COVID-19 pandemic.

We also observed relatively low use of the app-based diary in PrEPme. Mobile health interventions have been successful largely because of the convenience of the intervention within smartphone apps. However, PrEPme seemingly did not add value to the PCA intervention component. Studies suggest that aspects such as aesthetics, social networking ability, and gamification impact Black sexual minority men’s use of app-based HIV prevention apps [24,43]. The self-monitoring feature of the app-based diary could be refined for gamification and cultural responsiveness. PrEPme also did not maximize the power of health communication to tailor messaging. However, we are unable to identify reasons for nonuse. Qualitative insights or participant feedback could help in identifying barriers to app use. In light of previous analyses showing baseline effects on PRH [40], adding the mobile app–based diary may not be necessary in the presence of an effective PCA.

The intervention did observe relatively high proportions of willingness to accept PrEP referral and initiation, which could be attributed to the interpersonal dynamics between participants and the PCA [17]. Studies consistently show that peers and social networks are important, trusted sources of health information and effective interventionists among marginalized communities, including Black sexual minority men [16,38,44,45]. Studies also show that Black sexual minority men are more willing to initiate PrEP if their peers are using it. The usefulness of communicating with a PCA may not have been outweighed by the convenience of technology. PRH may not necessarily be the primary motivation for PrEP referral willingness or initiation among Black sexual minority men.

Despite the feasibility of this intervention, using a PCA to catalyze PRH and PrEP initiation among Black sexual minority men is not without challenges. DTD described internal conflicts regarding honoring participants’ disinterest in PrEP versus professional goals to increase uptake for HIV prevention in an autoethnography [17]. Additionally, managing discussions regarding side effects with Black sexual minority men whose health histories the PI or PCA was unfamiliar with or unqualified to discuss is important to consider in this peer-based approach. Concerns that being a PCA could overshadow professionalism as a researcher and health care professional were also salient [17]. PCAs should be trained to manage insider-outsider dynamics as an interventionist among Black sexual minority men, engage in active listening, and communicate with care [17,46]. Some qualities may not be able to be provided in training such as the shared social experiences and vulnerabilities of being Black sexual minority men.

### Limitations

Study limitations include insufficient sample size to detect effect sizes. Causal inferences cannot be drawn, and effectiveness cannot be established with a pre-post single-group design. It is also possible that unstudied external factors could have produced the changes observed. All data were self-reported. A larger trial is needed to definitively establish the effects of the intervention, including biological confirmation of PrEP use beyond self-report.

### Conclusions

Future research should test a statistically powered peer-based approach on PrEP initiation among Black sexual minority men. Psychometric tests should also be conducted to identify culturally relevant concepts of HIV risk and PrEP motivation for Black sexual minority men. Qualitative research should also clarify how app-based sexual risk diaries have unintended consequences of triggering self-stigma and shame versus informed decision-making [6]. Targeted studies among young Black sexual minority men younger than 35 years of age should be conducted, given their high HIV incidence and low PrEP uptake. Studies show age cohort differences regarding the needs and vulnerabilities among Black sexual minority men such that peers may be a more effective behavior change mechanism for younger men [33,47,48]. If effectively implemented, the person-centered approach of a PrEP-using PCA approach could lead to substantial community-level impact for Black sexual minority men because their needs are not the same.

## Abbreviations

ADAPT-ITT: Assessment, Decision, Administration, Production, Topical Experts, Integration, Training, and Testing
CHW: community health worker
MI: motivational interviewing
PCA: peer change agent
PI: principal investigator
PrEP: pre-exposure prophylaxis
PRH: perceived risk for HIV
